# Cardiovascular benefits and safety of sotagliflozin in type 2 diabetes mellitus patients with heart failure or cardiovascular risk factors: a bayesian network meta-analysis

**DOI:** 10.3389/fphar.2023.1303694

**Published:** 2023-11-17

**Authors:** Jiyifan Li, Chenyang Zhu, Jingru Liang, Jiarong Hu, Haiyang Liu, Zihan Wang, Ruifang Guan, Junwei Chow, Shiwei Yan, Longzhou Li, Fuyan Ma, Guo Ma

**Affiliations:** Department of Clinical Pharmacy, School of Pharmacy, Fudan University, Shanghai, China

**Keywords:** sotagliflozin, sodium-glucose co-transporters 2 inhibitor, type 2 diabetes mellitus, Bayesian network meta-analysis, heart failure, cardiovascular benefits, safety

## Abstract

**Background:** As an antidiabetic agent, sotagliflozin was recently approved for heart failure (HF). However, its cardiovascular benefits in type 2 diabetic mellitus (T2DM) patients with HF or cardiovascular (CV) risk factors have not been systematically evaluated. The aim of this study is to evaluate the cardiovascular benefits and safety of sotagliflozin in T2DM patients with HF or CV risk factors using Bayesian network meta-analysis.

**Methods:** Data were retrieved from PubMed, Embase, Web of Science, ClinicalTrials.gov, and Cochrane Library from their inception to 16 August 2023. Randomized controlled trials (RCTs) comparing sotagliflozin with a placebo, dapagliflozin, and empagliflozin in adult T2DM patients with HF or CV risks for at least 12 weeks were included in the study. Data analysis was conducted using R 4.2.3 and Stata 17.0. Cardiovascular efficacy outcomes included HF events (hospitalization or urgent visits for HF), MACE (deaths from CV causes, hospitalizations for HF, nonfatal myocardial infarctions, and strokes), cardiovascular death, the decrease in SBP, and weight loss. Safety outcomes are urinary tract infection, diarrhea, and diabetic ketoacidosis.

**Results:** Eleven studies with 30,952 patients were included. Compared to dapagliflozin and empagliflozin, 200 mg of sotagliflozin showed the best effect in reducing HF events [OR (95% CI), 0.79 (0.66, 0.94) and 0.90 (0.63, 1.27)]. Compared to dapagliflozin, 200 mg of sotagliflozin [OR (95% CI), 0.76 (0.66, 0.87)] was superior in preventing MACE. Compared to empagliflozin, 200 mg of sotagliflozin [OR (95% CI), 1.46 (1.04, 2.05)] was inferior in preventing CV death. Sotagliflozin showed a poorer SBP decreasing effect than empagliflozin and dapagliflozin [MD (95% CI), 1.30 (0.03, 2.56) and 2.25 (0.35, 4.14), respectively]. There was no significant difference between sotagliflozin and other interventions in weight loss. Sotagliflozin exhibited no increased risk for diabetic ketoacidosis or urinary tract infection among all interventions, however, it showed a mild risk for diarrhea than placebo [OR (95% CI), 1.47 (1.28, 1.69)].

**Conclusion:** Sotagliflozin displayed moderate CV benefits and acceptable safety. Sotagliflozin can be one of the recommended options for T2DM patients with HF or CV risk factors, which will be important for evidence-based use of sotagliflozin as well as decision-making of T2DM medication.

## 1 Introduction

As a chronic, irreversible metabolic disease, the prevalence of diabetes has increased sharply (from 4.6% to 9.8%) in the world in 20 years (International Diabetes Federation, 2021). Type 2 diabetes mellitus (T2DM) accounts for about 90% of diabetes (Ahmad et al., 2022). T2DM usually accompanies cardiovascular disease (CVD) such as heart failure (HF), and CVD is the leading cause of morbidity and mortality in T2DM (Joseph et al., 2022). Therefore, one of the main management goals of T2DM is to reduce the risk of cardiovascular (CV) adverse events by using antidiabetic agents (American Diabetes Association, 2021), e.g., sodium-glucose cotransporter 2 inhibitor (SGLT2i).

As a new class of antidiabetic agents, SGLT2i can reduce blood glucose concentration by inhibiting the reabsorption of sodium-glucose in proximal renal tubules and promoting urine sugar excretion. Multiple randomized controlled trials (RCTs) have confirmed that SGLT2i could exert a robust reduction of HF in T2DM adults and additional benefits of weight loss and blood pressure reduction (Leiter et al., 2014; Tikkanen et al., 2015; Wiviott et al., 2019). According to the 2021 American diabetes association (ADA) guidelines (American Diabetes Association, 2021), SGLT2i was recommended to treat T2DM patients with concurrent atherosclerotic cardiovascular disease (ASCVD) or its high-risk factors, HF, or chronic kidney disease. Two widely used SGLT2i, dapagliflozin and empagliflozin, have already been approved to treat HF with or without diabetes.

Sotagliflozin was the first dual SGLT1/2 inhibitor (SGLT1/2i) initially evaluated as an antidiabetic agent in 2019, and its adequate hypoglycemic effect was proved by a meta-analysis (Avgerinos et al., 2022). Subsequently, trials with CV outcomes consistently showed that it could reduce major CV events and hospitalization for HF (Bhatt et al., 2021a; Bhatt et al., 2021b; Cherney et al., 2021; 2023). Sotagliflozin was also approved to treat HF with or without diabetes by the U.S. Food and Drug Administration (FDA) in May 2023, making it a candidate for the recommended regimens for T2DM patients with HF or CV risks. In terms of safety, it was reported that sotagliflozin could increase the risk of ketoacidosis, urinary tract or genital tract infections, diarrhea, and volume depletion events (Musso et al., 2019; Zhou et al., 2022).

It is vital to evaluate the CV efficacy and safety of antidiabetic agents (e.g., sotagliflozin) in T2DM management and clinical drug selection using scientific methods including RCTs and network meta-analysis. However, there was only one RCT that had directly compared the CV benefits and safety of sotagliflozin and empagliflozin at present (Posch et al., 2022). The CV benefits of sotagliflozin in T2DM patients with HF or CV risks have not been systematically evaluated. When lacking direct comparisons, Bayesian network meta-analysis is an effective method to compare multiple treatments by combining direct and indirect evidence (Ahn and Kang, 2021). In this study, cardiovascular benefits and safety of sotagliflozin were evaluated by Bayesian network meta-analysis, which can provide relative rankings of sotagliflozin and other interventions, and will provide important evidence for rational use of sotagliflozin, empagliflozin, and dapagliflozin as well as drug-selection in T2DM patients with HF or CV risks.

## 2 Materials and methods

This study met the requirement of the Preferred Reporting Items for Systematic Reviews and Meta-Analyses (PRISMA) extension for reporting network meta-analysis (Hutton et al., 2015).

### 2.1 Search strategy

Five databases (PubMed, Embase, Web of Science, ClinicalTrials.gov, and Cochrane Library were searched for RCTs of sotagliflozin in the treatment of T2DM patients, with a time limit from database creation to 16 August 2023. The databases were searched with the following MeSH (Medical Subject Headings) terms: (1) Sotagliflozin OR LX-4211 [Title] AND (2) Diabetes Mellitus, Type 2 [MeSH] AND (3) Heart Failure [MeSH] OR Cardiovascular Diseases [MeSH] (Supplementary Table S2).

### 2.2 Eligibility criteria

The eligibility criteria conformed to the PICOS: (1) Population: adult T2DM patients with HF (with or without reduced LVEF) or existing CV risk factors (presence of ASCVD, dyslipidemia, hypertension, obesity, and so on); (2) Intervention: sotagliflozin (orally once-daily of 200 mg and 400 mg); (3) Comparison: SGLT2i (orally once-daily of dapagliflozin 10 mg, orally once-daily of empagliflozin 10 mg and 25 mg); (4) Outcomes: ①HF-events (hospitalizations or urgent visits for HF); ②major adverse cardiovascular events (MACE, including deaths from CV causes, hospitalizations for HF, nonfatal myocardial infarctions, and strokes) ③CV-death; ④decrease in systolic blood pressure (SBP); ⑤body weight (BW) loss; ⑥adverse events, i.e., urinary tract infection (UTI), diarrhea, and diabetic ketoacidosis (DKA); (S) Study type: RCTs. Secondary analyses and phase I studies were excluded. Additionally, hypoglycemic indicators were not evaluated for insufficient data posted by relevant RCTs.

### 2.3 Data extraction and risk of bias assessment

Data was recorded under the following headings: (1) study profile (title, first author, publication year, study design); (2) baseline characteristics (age, sex, BW, SBP, disease, and so on); (3) predefined outcomes. The risk of bias was assessed with the Cochrane risk of bias tool, including random sequence generation, allocation concealment, performance and detection bias, incomplete outcome data, and selective reporting. Studies were included after a blinded review by two independent reviewers (i.e., Li and Zhu), and referral to a third independent reviewer (i.e., Liang) in case of disagreement.

### 2.4 Heterogeneity, model fit, and inconsistency assessment

Cochran’s I^2^ test was used to assess heterogeneity. When I^2^ <50%, the heterogeneity is not obvious, and fixed-effect models should be applied. Model fit was assessed through the deviance information criterion (DIC), and there is no evidence of inconsistency if the difference between the inconsistency and consistency models is under 5, thus the simpler model with a smaller DIC should be used (Spiegelhalter et al., 2002; Daly et al., 2022). Node-splitting approaches are needed when loops are formed by two or more studies. Markov Chain Monte Carlo algorithm was used for each outcome after 100,000 simulation iterations, 50,000 adaptation iterations, and a thinning interval of 1.

### 2.5 Data synthesis and analysis

A network meta-analysis was performed with a Bayesian approach using R version 4.2.3 with the GEMTC, JAGS, and MULTINMA packages, and Stata 17.0. Pooled odds ratios (OR) with 95% CIs for dichotomous outcomes and pooled mean differences (MD) with 95% CIs for continuous outcomes were calculated and shown in league tables. The surface under the cumulative ranking curve (SUCRA) was also calculated to rank the effectiveness and safety of each treatment, and a higher SUCRA value indicates a better or safer treatment.

### 2.6 Publication bias assessment

Publication bias is a common phenomenon in clinical literature in which positive results have a better chance of being published (Dubben and Beck-Bornholdt, 2005), and it is thus caused by unpublished or unnoticed studies. Analysis of publication bias was carried out using Stata 17.0. The comparison-adjusted funnel plots for assessment of publication bias were recommended for network meta-analysis (Higgins et al., 2021). The visually symmetric distribution of scattered dots about the combined effect size line (the central vertical line of the funnel) indicates that the publication bias was not significant.

## 3 Results

### 3.1 Study selection and characteristics

**FIGURE 1:**
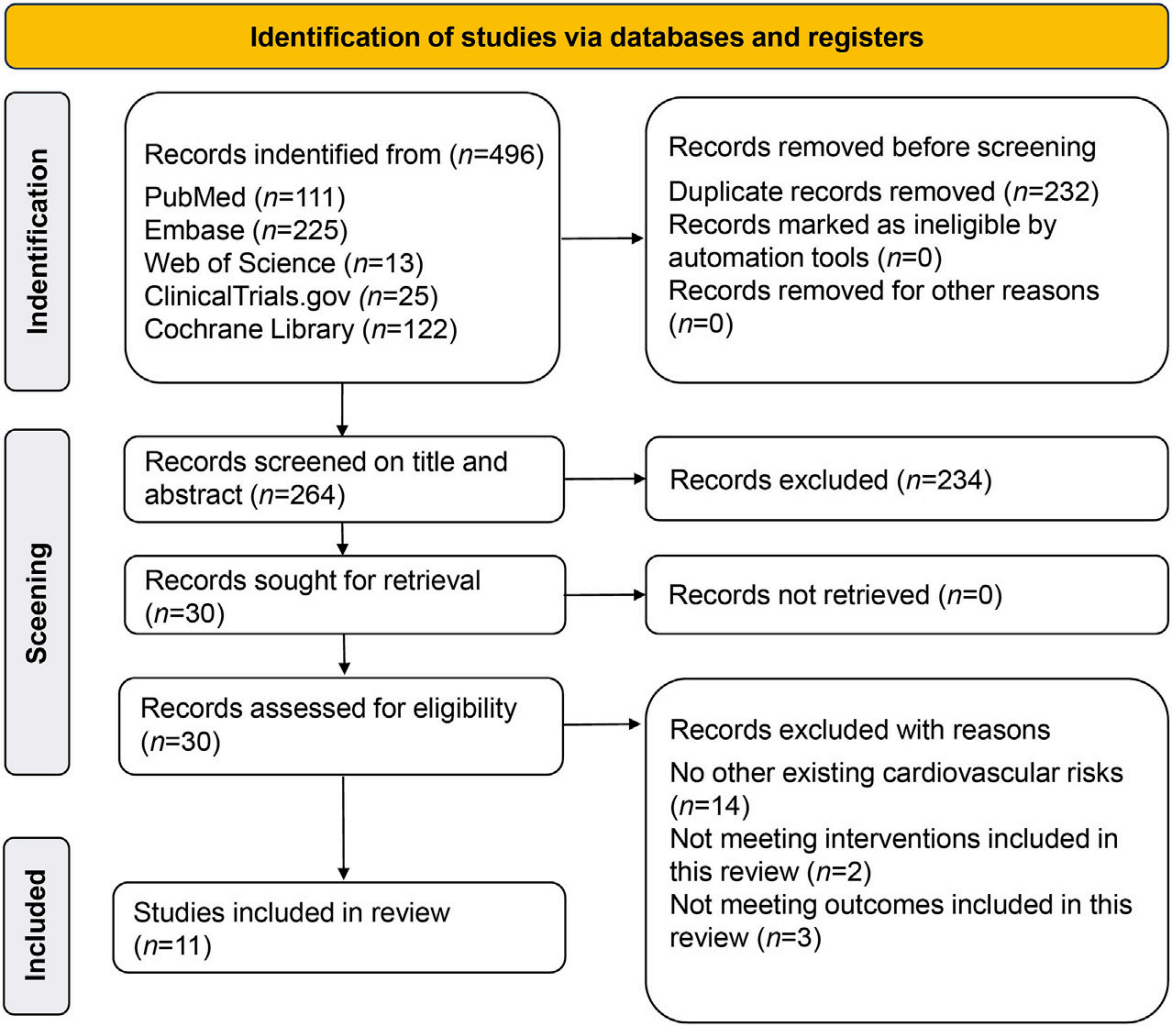
PRISMA flow chart of Study Selection Process.

**FIGURE 2:**
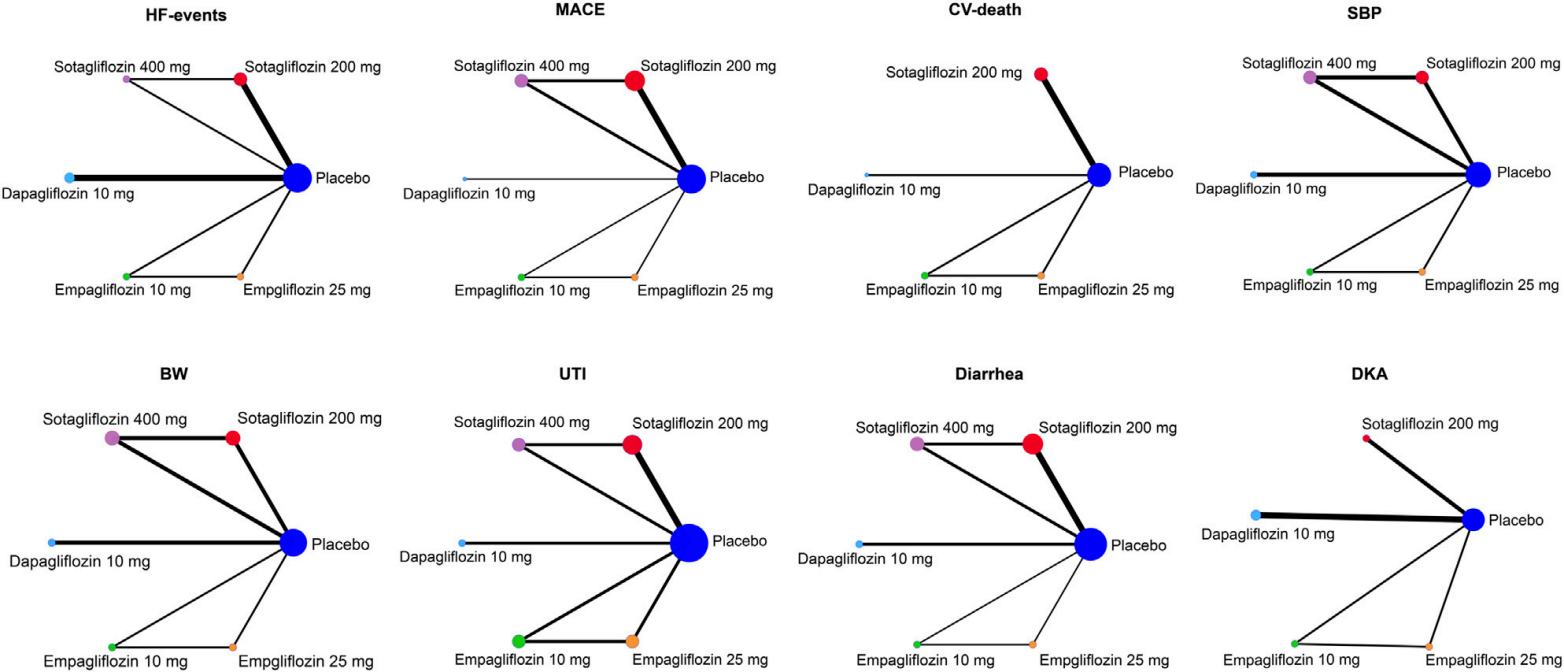
Network plot of cardiovascular efficacy and safety outcomes.

A total of 496 studies were retrieved; 232 duplicates were screened out, 224 studies were excluded after reading the titles and abstracts, and 19 studies were excluded for not meeting inclusion criteria; this left 11 RCTs with 30,952 participants which were finally included (Leiter et al., 2014; Tikkanen et al., 2015; Zinman et al., 2015; McMurray et al., 2019; Phrommintikul et al., 2019; Wiviott et al., 2019; Bhatt et al., 2021a; Bhatt et al., 2021b; Cherney et al., 2021; 2023; Solomon et al., 2022). The PRISMA flow chart is shown in Figure 1. The weighted means of age, BMI, and baseline SBP were 66.2 years, 30.3 kg/m^2^, 132.1 mmHg, respectively, and the detailed study characteristics were listed in Supplementary Table S1. The network plots of each outcome are illustrated in Figure 2.

### 3.2 Risk of bias assessment

**FIGURE 3:**
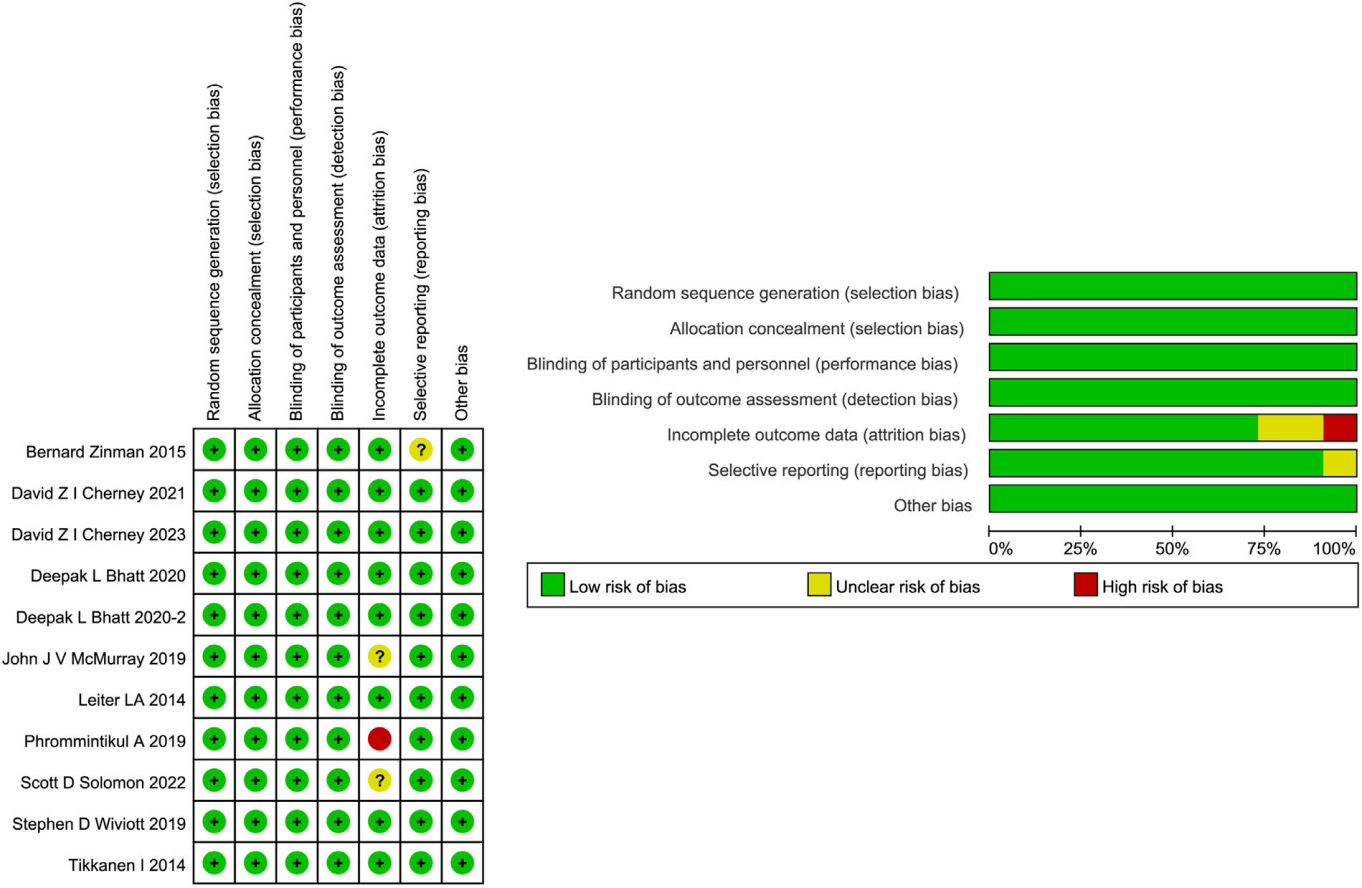
Risk of bias assessment of the included studies.

All 11 studies were identified as at low risks of selection and performance bias. However, one study (Phrommintikul et al., 2019) was deemed as having a high risk for incomplete outcome data because no drug safety outcomes were reported. Two studies (McMurray et al., 2019; Solomon et al., 2022) are rated as unclear risks for possibly incomplete safety results. One study (Zinman et al., 2015) was evaluated as at unclear risk for reporting bias due to incomplete baseline information. The detailed risk of bias assessment is shown in Figure 3. Overall, quality was appraised as being of a moderate level.

### 3.3 Cardiovascular Efficacy outcomes

#### 3.3.1 HF-events

**FIGURE 4:**
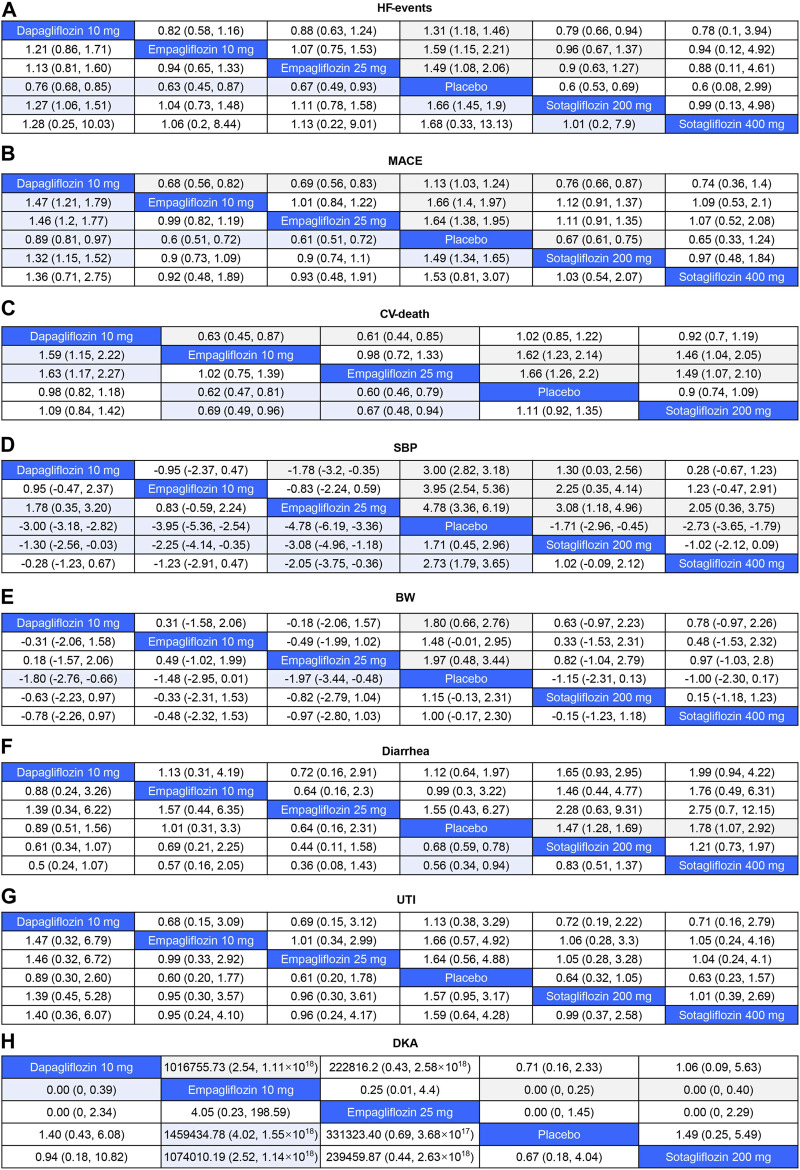
League Table for Eight Outcome Measures The colored cells indicated that the results were significant. For odds ratios (OR) in each cell, OR<1 favors column-defining treatment. For mean difference (MD), MD < 0 favors column-defining treatment. HF-events, hospitalizations or urgent visits for heart failure; major adverse cardiovascular events (MACE), including deaths from CV causes, hospitalizations for heart failure, nonfatal myocardial infarctions, and strokes; CV-death, death for cardiovascular causes; SBP, the decrease in systolic blood pressure; BW, body weight loss; UTI, urinary tract infection; DKA, diabetic ketoacidosis. **(A)**, HF-events; **(B)**, MACE; **(C)**, CV-death; **(D)**, SBP; **(E)**, BW; **(F)**, Diarrhea; **(G)**, UTI; **(H)**, DKA.

**FIGURE 5:**
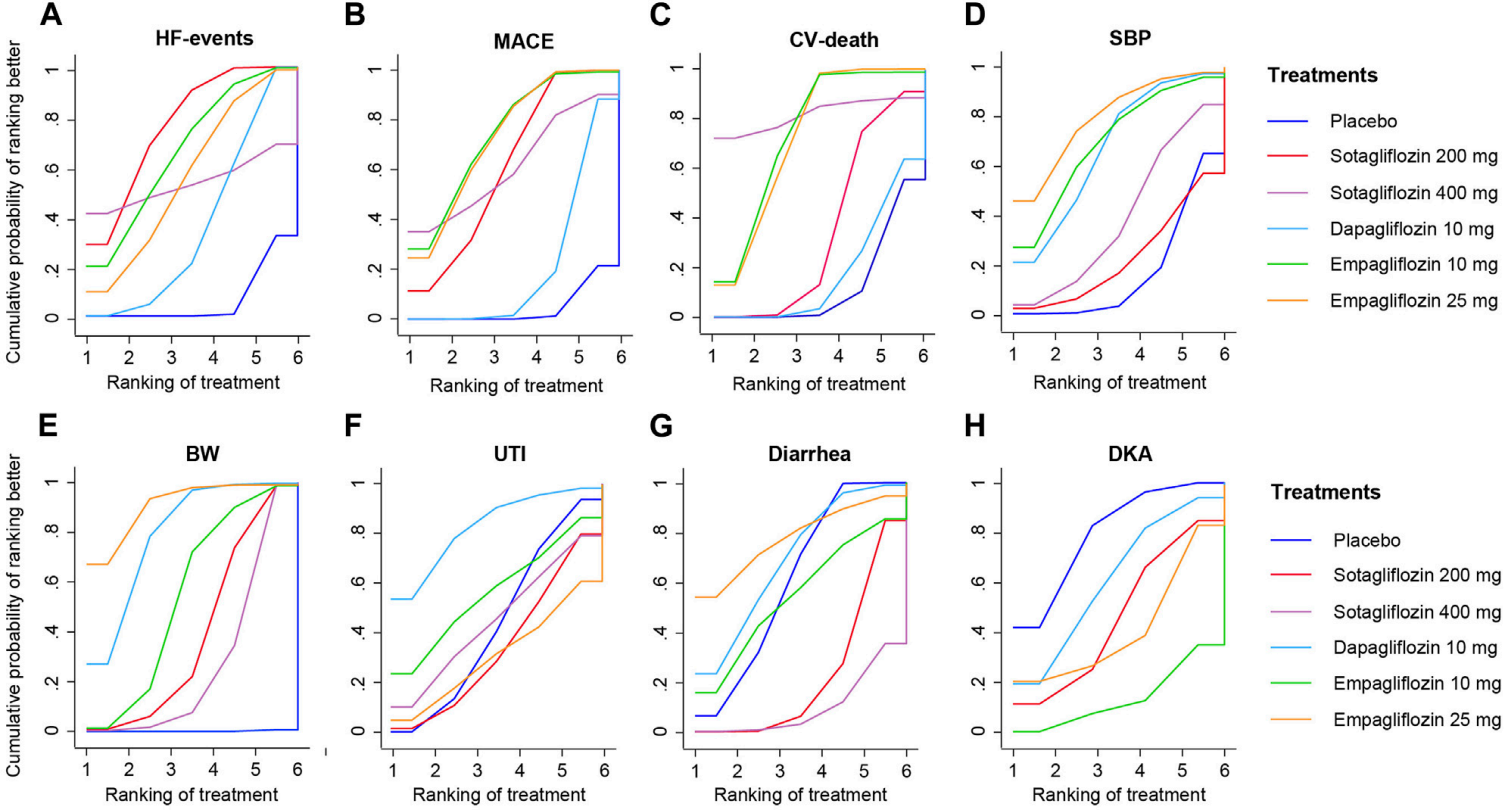
SUCRA plot for Eight Outcome Measures. **(A)**, HF-events; **(B)**, MACE; **(C)**, CV-death; **(D)**, SBP; **(E)**, BW; **(F)**, Diarrhea; **(G)**, UTI; **(H)**, DKA.

As shown in Figure 4A, 200 mg of sotagliflozin showed superior effect in reducing hospitalization or urgent visits for HF than 10 mg of dapagliflozin (OR, [95% CI], 0.79 [0.66, 0.94]), and showed no significant difference compared with 10 mg and 25 mg of empagliflozin (OR, [95% CI], 0.96 [0.67, 1.37], 0.90 [0.63, 1.27], respectively). There was no significant dose-response relationship between 200 mg and 400 mg of sotagliflozin (OR, [95% CI], 1.01 [0.20, 7.90]). The results of SUCRA (Figure 5A; Table 1) indicated that 200 mg of sotagliflozin exhibited the best effect in reducing hospitalization or urgent visits for HF (77.5%), followed by 10 mg of empagliflozin, 25 mg of empagliflozin, 400 mg of sotagliflozin and 10 mg of dapagliflozin (67.3%, 57.3%, 53.8%, and 37.5%, respectively).

**TABLE 1:** SUCRA (%) of various interventions for cardiovascular efficacy and safety outcomes.

Interventions		PLA	SOT200	SOT400	DAP10	EMP10	EMP25
**HF-events**	SUCRA(%)	6.6	77.5	53.8	37.5	67.3	57.3
	Rank	6	1	4	5	2	3
**MACE**	SUCRA(%)	4.5	62.0	62.2	21.8	75.6	73.8
	Rank	6	4	3	5	1	2
**CV-death**	SUCRA(%)	14.4	40.6	NA	20.6	85.7	88.7
	Rank	5	3	NA	4	2	1
**SBP**	SUCRA(%)	17.6	23.0	40.1	69.1	70.3	80.0
	Rank	6	5	4	3	1	2
**BW**	SUCRA(%)	0.1	41.3	28.7	80.6	57.1	92.2
	Rank	6	4	5	2	3	1
**UTI**	SUCRA(%)	44.9	35.3	46.1	83.8	57.6	32.4
	Rank	4	5	3	1	2	6
**Diarrhea**	SUCRA(%)	61.9	23.7	10.3	70.1	55.3	78.7
	Rank	3	5	NA	2	4	1
**DKA**	SUCRA(%)	80.4	47.4	NA	62.3	16.1	43.7
	Rank	1	3	NA	2	5	4

SUCRA, the surface under the cumulative ranking curve value; PLA, placebo; SOT200, sotagliflozin once-daily of 200 mg; SOT400, sotagliflozin once-daily of 400 mg; DAP10, dapagliflozin once-daily of 10 mg; EMP10, empagliflozin once-daily of 10 mg; EMP25, empagliflozin once-daily of 25 mg; HF-events, hospitalizations or urgent visits for heart failure; major adverse cardiovascular events (MACE), including deaths from CV, causes, hospitalizations for heart failure, nonfatal myocardial infarctions, and strokes; CV-death, death for cardiovascular causes; SBP, the decrease in systolic blood pressure; BW, weight loss; UTI, urinary tract infection; DKA, diabetic ketoacidosis; NA, not available. A higher rank with a larger SUCRA value indicates that the intervention is better for each of the outcomes.

#### 3.3.2 MACE

As shown in Figure 4B mg of sotagliflozin showed better effect in reducing all MACEs than 10 mg of dapagliflozin (OR, [95% CI], 0.76 [0.66, 0.87], and showed no significantly poorer effect than 10 mg and 25 mg of empagliflozin (OR, [95% CI], 1.12 [0.91, 1.37], 1.11 [0.91, 1.35], respectively). The results of SUCRA (Figure 5B; Table 1) indicated that 10 mg and 25 mg of empagliflozin exhibited superior effects in reducing MACEs (75.6% and 73.8%, respectively), followed by 400 mg, 200 mg of sotagliflozin, and 10 mg of dapagliflozin (62.2%, 62.0%, and 21.8%, respectively).

#### 3.3.3 CV-death

Sotagliflozin showed a poorer effect than 10 mg and 25 mg of empagliflozin (OR, [95% CI], 1.46 [1.04, 2.05], 1.49 [1.07, 2.1], respectively) (Figure 4C) in reducing CV-death. Although the SUCRA (Figure 5C; Table 1) showed that, 200 mg of sotagliflozin (40.6%) ranked higher than 10 mg of dapagliflozin (20.6%) in reducing CV-death, there was no significant difference (OR, [95% CI], 0.92 [0.70, 1.19]). The results of 400 mg of sotagliflozin were not evaluated for heterogeneity.

#### 3.3.4 Blood pressure control

Compared with placebo, 200 mg and 400 mg of sotagliflozin showed significant SBP reduction (OR, [95% CI], −1.71 [-2.96, −0.45], −2.73 [-3.65, −1.79]) (Figure 4D), but they showed the poorest efficacy among all the included interventions in SUCRA (23.0%, 40.1%) (Figure 5D; Table 1). 400 mg of sotagliflozin failed to outperform 10 mg of dapagliflozin and 10 mg of empagliflozin in SBP reduction (OR, [95% CI], 0.28 [-0.67, 1.23], 1.23 [-0.47, 2.91], respectively).

#### 3.3.5 Body weight loss

Compared with other interventions, sotagliflozin did not show a significant body weight loss effect (Figure 4E). The results of SUCRA (Figure 5E; Table 1) indicated that 25 mg of empagliflozin exhibited the best effect in body weight loss (92.2%), followed by 10 mg of dapagliflozin, 10 mg of empagliflozin, 200 mg and 400 mg of sotagliflozin (80.6%, 57.1%, 41.3%, 28.7%, respectively).

### 3.4 Safety outcomes

As shown in Figure 4F, all the included interventions showed no significantly higher risk for UTI. Compared with placebo, 200 mg and 400 mg of sotagliflozin increased the incidence of diarrhea (OR, [95% CI], 1.47 [1.28, 1.69], 1.78 [1.07, 2.92], respectively), but the other interventions exhibited no significant risk for diarrhea (Figure 4G). Compared with placebo, sotagliflozin was not associated with significant risks of DKA (OR, [95% CI], 1.49 [0.25, 5.49]), and the other interventions showed similar outcomes (Figure 4H **)**. The SUCRA values of safety outcomes (Table 1) indicated that 10 mg of dapagliflozin (83.8%) showed the lowest risks while 25 mg of empagliflozin (32.4%) showed the highest risks in UTI; 10 mg of dapagliflozin (62.3%) showed the lowest risks while 10 mg of empagliflozin (16.1%) showed the highest risks in DKA; and 25 mg of dapagliflozin (78.7%) showed the lowest risks while 400 mg of sotagliflozin (10.3%) showed the highest risks in diarrhea.

### 3.5 Inconsistency and heterogeneity tests

The difference values of DIC between inconsistency and consistency models in all eight outcomes were less than five (Supplementary Table S3), thus consistency models were applied. For most comparisons, the heterogeneity was not significant. Sensitivity analysis was conducted, and the I^2^ value of the comparison between placebo and sotagliflozin (200 mg) in CV-death decreased from 86.7% to 0% after eliminating one study (Bhatt et al., 2021a). However, for the following comparisons, heterogeneity could not be decreased because there were only two studies included for each comparison, i.e., placebo versus dapagliflozin in SBP, BW, and UTI; placebo versus 400 mg of sotagliflozin in UTI and DKA, and 200 mg sotagliflozin versus 400 mg of sotagliflozin in SBP (Supplementary Table S4). Therefore, a random-effect model was used.

### 3.6 Publication bias

**FIGURE 6:**
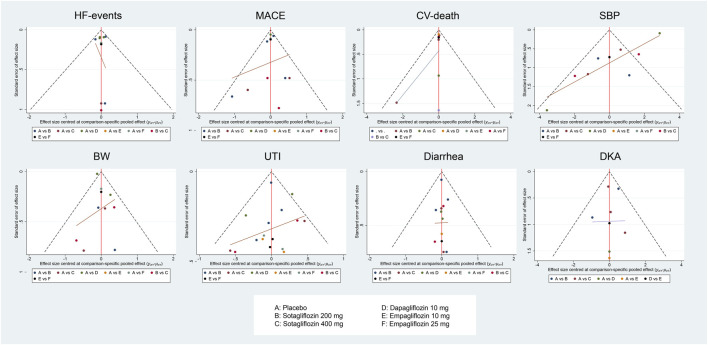
Figure 4A, HF-events; Figure 4B, MACE; Figure 4C, CV-death; Figure 4D, SBP; Figure 4E, BW; Figure 4F, Diarrhea; Figure 4G, UTI; Figure 4H, DKA. Comparison-adjusted funnel plot for eight outcomes.

The comparison-adjusted funnel plots (Figure 6) suggested that publication bias may exist in CV-death and SBP in the analysis. However, there were fewer than ten studies included in each of the outcomes, so publication bias assessment using funnel plots might not be reliable (Ioannidis and Trikalinos, 2007; Sterne et al., 2011; Dalton et al., 2016).

## 4 Discussion

In the present network meta-analysis, CV benefits and safety of sotagliflozin, as well as dapagliflozin and empagliflozin, were evaluated in T2DM with HF or CV risks. Overall, sotagliflozin, dapagliflozin, and empagliflozin displayed differentiated CV benefits and acceptable safety. Among them, sotagliflozin displayed the best efficacy in avoiding HF hospitalization or urgent visits, moderate effect in reducing MACEs including nonfatal myocardial infarction, strokes, etc., and no superior effect over other interventions in preventing CV-death. A dose-response relationship was not obvious in 200 mg and 400 mg of sotagliflozin, nor as in 10 mg and 25 mg of empagliflozin in terms of CV benefits. Additionally, empagliflozin displayed the best effect in reducing MACE and CV-death, however, only two eligible studies on empagliflozin were included (Tikkanen et al., 2015; Zinman et al., 2015), and further studies are needed to prove its superiority.

Sotagliflozin, dapagliflozin, and empagliflozin were reported to have risks of DKA, UTI, and genital tract infections (Vasilakou et al., 2013; Rosenstock and Ferrannini, 2015; Rieg and Vallon, 2018), however, this study found that there was no significant risk of DKA or UTI among all the included interventions. Generally speaking, dapagliflozin exhibited the best safety, followed by sotagliflozin and empagliflozin according to the SUCRA values. Additionally, only sotagliflozin showed mild risks for diarrhea among all treatments, which was consistent with a previous meta-analysis (Avgerinos et al., 2022). The sotagliflozin-induced diarrhea may be due to its inhibition of SGLT1 (Tsimihodimos et al., 2018), which was associated with the osmotic diarrhea caused by the non-absorbed excess of glucose and galactose in the intestinal lumen (Song et al., 2016).

There was only one published frequentist network meta-analysis of SGLT1/2i versus SGLT2i (Teo et al., 2022). This study conducted a thorough comparison of a variety of SGLT1/2i and SGLT2i with multiple outcomes (i.e., CV, metabolic, and renal indicators) in type 1 and type 2 diabetic patients with or without HF or CV risks. Nevertheless, diabetes without CV risks is not the main indication of SGLT2i or SGLT1/2i according to the ADA guidelines. Consequently, our study refined the inclusion criteria to better meet the guidelines. Furthermore, a frequentist network meta-analysis is generally considered less promising than a Bayesian network meta-analysis in evaluating performance measures in sparse networks (Seide et al., 2020). Therefore, the Bayesian approach was selected in our study.

## 5 Limitation

In the present study, we evaluated the cardiovascular benefits and safety of sotagliflozin in T2DM patients with HF or CV risk factors using outcome measures, i.e., HF events, MACE, CV death, decrease in SBP and BW, but did not provide any hypoglycemic outcome measures, e.g., glycated hemoglobin (HbA1c). The reason was that only one eligible RCT (Cherney et al., 2021) on sotagliflozin in treating T2DM with HF or CV risk factors had provided any hypoglycemic outcomes. Moreover, the hypoglycemic effect of sotagliflozin has been assessed in previous meta-analyses (Teo et al., 2022) and RCTs (Lexicon Pharmaceuticals, 2021a; Lexicon Pharmaceuticals, 2021b).

HF events are considered as a strong HF and CV prognostic factor, and occurred in 61.3% of HF patients (Chun et al., 2012). Blood pressure control is considered an important factor in HF and CV management (Lee et al., 2022). Thus, both HF events and SBP were included in this study. Nevertheless, HF events do not cover all the prognostic outcome measures of HF, other outcome measures (e.g., B-type natriuretic peptide (BNP), N-terminal pro-BNP (NT-proBNP) level, estimated glomerular filtration rate (eGFR), and ejection fractions) should be included. However, only one study on sotagliflozin (Cherney et al., 2021) reported other HF prognostic outcome measures (i.e., eGFR). Consequently, other prognostic outcomes of HF were not included in the network meta-analysis.

Among all the eligible studies, there were only four publications that reported on sotagliflozin, five studies on dapagliflozin, and two studies on empagliflozin in treating T2DM patients with HF or CV risks, indicating more RCTs on this population need to be conducted so as to reach a solid conclusion. Moreover, a strict search, data selection, and sensitivity analysis were conducted, however, heterogeneity in SBP, BW, UTI, and DKA, and publication bias of CV-death and SBP were not avoided.

Duration of diabetes plays an important role in evaluating CV risks in T2DM patients. An approximately 20% increased risk was associated with every five-year increment in the duration of diabetes (de Jong et al., 2022). Therefore, if the patients without HF or CV risks had been diagnosed with diabetes for long enough, they also met the inclusion criteria. Sub-group analysis on the duration of diabetes could be conducted if only the RCTs had provided more detailed baseline characteristics. More head-to-head RCTs targeting this high-CV risk diabetic population with details on the duration of diabetes need to be conducted so as to provide more reliable evidence on the CV benefits and safety of sotagliflozin. T2DM easily results in diabetic nephropathy, and diabetic nephropathy further increases the risk of HF and ischemic events (Udell et al., 2015). Consequently, outcomes regarding renal functions should also be integrated into RCT designed for T2DM patients with CV risks.

## 6 Conclusion

Sotagliflozin is optional in treating T2DM patients with HF or CV risk factors and displayed moderate CV benefits and acceptable safety. Overall, empagliflozin and sotagliflozin were superior to dapagliflozin in CV benefits, while dapagliflozin was superior to empagliflozin and sotagliflozin in safety.

## Data Availability

The original contributions presented in the study are included in the article/Supplementary Material, further inquiries can be directed to the corresponding author.

## Abbreviations

T2DM, type 2 diabetic mellitus; RCT, randomized controlled trial; SGLT, sodium-glucose co-transporters; SGLT2i, sodium-glucose co-transporters 2 inhibitors; HF, heart failure; CV, cardiovascular; PLA, Placebo; SOT200, once-daily of 200 mg sotagliflozin; SOT400, once-daily of 400 mg sotagliflozin; EMP10, once-daily of 10 mg empagliflozin; EMP25, once-daily of 25 mg empagliflozin; DAP10, once-daily of 10 mg dapagliflozin; HF-events, hospitalization or urgent visit for heart failure; MACE, major adverse cardiovascular events, including deaths from CV causes, hospitalizations for HF, nonfatal myocardial infarctions, and strokes; CV-death, death from cardiovascular causes; SBP, decrease in systolic blood pressure (mm/Hg); BW, decrease in body weight (kg); UTI, urinary tract infection; DKA, incidents of diabetic ketoacidosis; NA, not available.
